# Metastable Features of Economic Networks and Responses to Exogenous Shocks

**DOI:** 10.1371/journal.pone.0160363

**Published:** 2016-10-05

**Authors:** Ali Hosseiny, Mohammad Bahrami, Antonio Palestrini, Mauro Gallegati

**Affiliations:** 1 Department of Physics, Shahid Beheshti University, G.C., Evin, Tehran 19839, Iran; 2 Department of Economics, Università Politecnica delle Marche, Ancona, Italy; East China University of Science and Technology, CHINA

## Abstract

It is well known that a network structure plays an important role in addressing a collective behavior. In this paper we study a network of firms and corporations for addressing metastable features in an Ising based model. In our model we observe that if in a recession the government imposes a demand shock to stimulate the network, metastable features shape its response. Actually we find that there exists a minimum bound where any demand shock with a size below it is unable to trigger the market out of recession. We then investigate the impact of network characteristics on this minimum bound. We surprisingly observe that in a Watts-Strogatz network, although the minimum bound depends on the average of the degrees, when translated into the language of economics, such a bound is independent of the average degrees. This bound is about 0.44ΔGDP, where ΔGDP is the gap of GDP between recession and expansion. We examine our suggestions for the cases of the United States and the European Union in the recent recession, and compare them with the imposed stimulations. While the stimulation in the US has been above our threshold, in the EU it has been far below our threshold. Beside providing a minimum bound for a successful stimulation, our study on the metastable features suggests that in the time of crisis there is a “golden time passage” in which the minimum bound for successful stimulation can be much lower. Hence, our study strongly suggests stimulations to arise within this time passage.

## Introduction

Addressing the causes of business cycles and its dynamics is a major goal in economics. Clearly, studying network structure can reveal some unknown features in this regard. It has been shown for example that the topology of the macroeconomic networks can have serious impacts on the cascade of crises in a world-wide scheme [1]. It has been proved that countries with higher connectivity in intersectoral collaborations have more serious avalanches in the time of crisis [2]. In regular interaction of systems, central limit theorem states that when the number of random variables grow, the fluctuations would overlap and dampen each other effectively. It however has been proved that in the network of production, it is the topology of the network that addresses constructive or destructive fluctuation effects [3]. While some networks are robust to random fluctuations, some other are vulnerable. A big deal of attention in the literature has been devoted to finding out how networks of production are vulnerable to the propagation of crisis. In the present work, on the contrary, we study the response of the network of firms to a demand shock together with the recovery acts imposed by policy makers.

In the Great Recession which occurred recently, amongst economists who favored the stimulation policies, some believed that in order to recover the economy, the stimulation must be huge. Such stimulation was favored especially because the economy was experiencing a near zero short-term nominal interest which limited the Central Bank’s ability to stimulate economic growth, see e.g. [4] and [5]. In such an extreme situation, these economists suggested that a small stimulation fails to help the economy experience fast recovery. In this paper through studying an Ising model of networks of firms, we investigate the metastability features of the network and its response to a demand shock. We actually evaluate the existence of the minimum bound for the size of a successful fiscal stimulation and its relation to the production output.

There can be different forms of fiscal stimulation such as tax cut and government spending. In the context of the present study we suppose that the government only purchases from firms and corporations in a fiscal stimulation. Actually a tax cut itself will be an indirect purchase from firms and corporations.

The Ising model has been proposed as a simple model to describe networks of firms (see for example Brock et al. (2001) [6] and Durlauf et al. (2010) [7]). The proposal is as follows. In a production network, each firm is connected to some other firms. Firms and corporations can buy each other’s products as intermediate goods or services. Now, each firm has a maximum and minimum capacity of production. Each firm possesses a minimum level where production below it results in loss rather than profit. Besides, each firm has a maximum capacity of production where producing over that is impossible. Each firm can sell its products as intermediate goods or services to its neighbors in the network. Now, for a firm that its neighbors work with their maximum capacity, it is more likely that orders are high enough to allow working with maximum capacity. As well, if the neighbors work with minimum capacity, it is likely that this firm works with its minimum capacity. So, firms force their neighbors to have a status similar to themselves. The situation is however stochastic and one can only talk about probability. If your neighbors do not buy your total products, you can still work with your maximum capacity and keep your extra production as inventory investment. This possibility is however limited.

If we want to model the interaction of firms in a network, we can simplify it as much as possible. For the simplest model, we can think of firms with a bi-status situation. Each firm can choose either its maximum production level or its minimum production level; a thing that we may indicate by up and down status. In a network of firms, if your neighbors choose an up status, they force you to choose an up status and vice versa. So, a network of firms at its simplest approximation can be considered as an Ising model. For a comprehensive discussion see [6, 7].

A collection of firms in an interacting network is subject to a collective behavior accompanied by an emergent phenomena. In a Keynesian economy it is believed that in depression where unemployment is high, the agents themselves reduce consumption. As a result, the Keynesian school suggests intervention of governments for the recovery of economy. If we consider an Ising model as a model to describe economy, we can read the Keynesian view of depression as the behavior of an Ising model below the critical temperature. Below the critical temperature, we have symmetry breaking. When a maximum of firms choose to work with minimum capacity, without a shock such as government stimulation, it is unlikely that the majority would decide to change mind and work with maximum capacity. So, if we study networks of firms through an Ising model, to simulate large scale crises within the Keynesian framework, we should consider the model under temperatures below the critical temperature.

To find if there is a minimum bound for fiscal stimulation in economy, we consider metastable features of an Ising model. For an Ising network below the critical temperature, when a majority of dipoles have chosen a downward direction, in order to stimulate them to choose an upward direction, you need to impose an external magnetic field. Theoretically, we believe that even with the effect of very weak stimulating fields, the system changes its status and moves from a downward direction to upward. The process however might be time consuming. If the stimulating field is very weak, for a long period of time, spins may not change their direction to upward. That is why we call these states, metastable states. If in our problem we are concerned about time (as we really are in economy), then the intensity of stimulating fields actually matters. In this paper we review studies concerning metastable features of the Ising model before translating the results of such studies to the macroeconomy language. We then run simulations concerning both cubic and small world networks to find the desired minimum bound.

Metastable features of the Ising model have been widely studied in physics. For studies concerning kinetic Ising see [8]. The life time of metastable states has been worked out in [9–13]. Understanding the kinetic behavior of the matter utilizing the droplet theory was considered in [14]. Dynamic phase transition was studied to understand some critical features of the matter, see [15–17] for details. For a study on the response of the model to an impulse stimulating field see [18] and [19]. For a review on droplet theory and dynamics of the Ising model see [20, 21].

Statistical physics has taught us that the path from micro to macro is not straightforward. This is why heterogeneous agent based models are studied (see for example [22–26]). Ising model has been applied widely in this framework (see for example [27]). Critical phenomena made it clear that network structure can play an important role to address aggregate behaviour. Currently many different issues are being studied via network glasses [28–36].

In this paper, trying to understand metastable features of the network of firms and corporations in economy, we first translate the desired parameters in economy to the parameters in the Ising model. We then review studies on the life time of the metastable states and direct our analysis towards the appropriate domain for simulation. We first check if there is a minimum bound for a successful fiscal stimulation in an Ising approximation of networks of firms in two dimensional cubic lattice. We then search for such a minimum bound in a small world network. Finally, we consider our findings and try to have an examination for economies of the US and EU in 2009 when a stimulation policy were to be imposed. Surprisingly, in spite of the simplification, the model suggests a reasonable bound.

## Materials and Methods

### Metastable Features in an Ising Model

**Fig 1:**
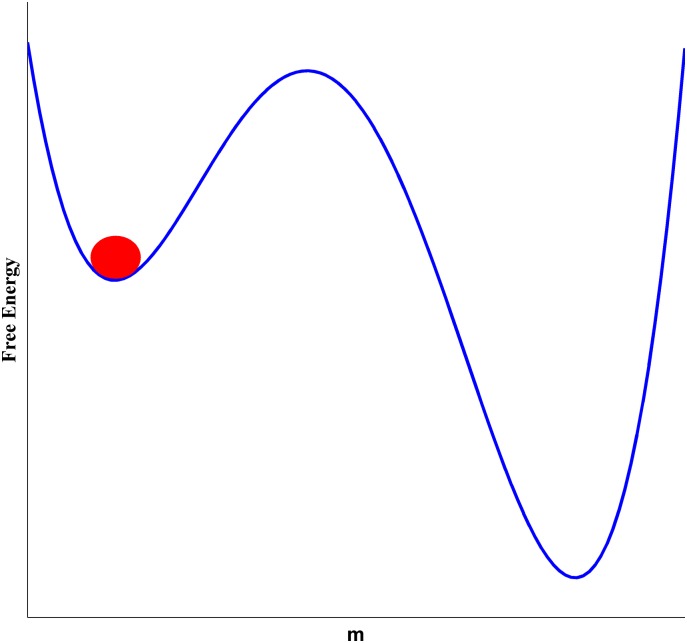
Below the critical temperature, the system is in its minimum with *m* ≈ −1. By imposing a weak upward external field, although the symmetry breaks and theoretically the system should move to its global minimum, in fact it should pass a local maximum. In such cases transitions are time consuming.

In the Ising model metastable features have been widely studied. Consider an Ising model with Hamiltonian

$$\begin{array}{c} H=-\beta J\Sigma_{<ij>}S_{i}.S_{j}-\beta H\Sigma_{i}S_{i}, \end{array}$$ (1)

where *J* represents the interaction of neighbors, and *H* indicates the external field. In the absence of an external field, and below the critical temperature, the symmetry is broken and the system finds a non-zero magnetization. This means that the majority of spins choose the same direction, say a downward direction. Now, if we impose an external field in the opposite direction (say upward), from the theoretical point of view the majority of spins should flip upward putting the system into its other vacuum. Although theoretically, in the presence of an external field the free energy is no more symmetrical, and the system should fall into its global minimum, but the process is still time consuming. Now let’s see how it works. From a free energy point of view, after imposing an upward magnetic field, the global minimum would be around *m* ≈ 1, see Fig 1. If we look at micro levels however things are different.

**Fig 2:**
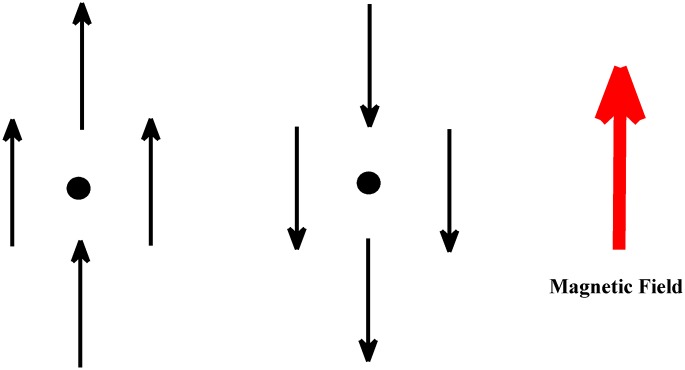
While the direction of all spins are downward, we have imposed a stimulating upward field. Theoretically, if all spins turn upward, the level of energy will be minimum. In the meantime however if the external field is weak, each spin needs to overcome forces by its neighbors and go to a higher local level of energy. This is not a favorable transition for each spin. For strong fields things are different. If the stimulating field is about 8*J*, the spin feels that all neighbors are upward and flip easily.

At microlevel, for a chosen spin, in average the neighbors are downward. If the stimulating field is weaker than 4*J*, choosing an upward direction for the spin would result in having a higher level of energy, see Fig 2. Due to the Boltzmann energy probability, the spin is unwilling to comply with the external field. In the language of game theory, it is similar to the prisoner dilemma problem. If all spins choose an upward direction, the total energy will be lower and every dipole would be better off. For a sole spin however flipping upward is to get in a higher level of energy causing an unwanted situation. In a network of firms in recession, if all firms simultaneously decide to hire new labors and work with maximum capacity, everybody is better off. For a single firm, starting production with maximum capacity while orders are in minimum level is a risky act that most probably results in loss. From a Keynesian point of view a government stimulating policy similar to a magnetic field helps economy to escape from its unwanted minimum to a favorable one.

Let’s go back to our Ising model. After imposing a magnetic field, gradually more and more spins flip upward. In our problem we are concerned with the lifetime of the metastable state. Suppose that below the critical temperature our system has chosen the downward direction with magnetization per site close to −1. If a stimulating upward magnetic field with a low intensity *H* is imposed, since the force experienced by each spin due to its neighbors is bigger than the stimulating field, the chance for the spin direction to flip is low. As time goes by, however gradually the stimulating field manages to flip more and more spins upward. When half of the spins are upward, in average for each spin, the forces of neighbors cancel out, outing us from a metastable trap. In this situation our stimulating field easily forces the spins to take an upward direction. The period that it takes for the stimulating field to drive magnetization from its initial value to a zero value is called the metastable lifetime denoted here by *τ*.

**Fig 3:**
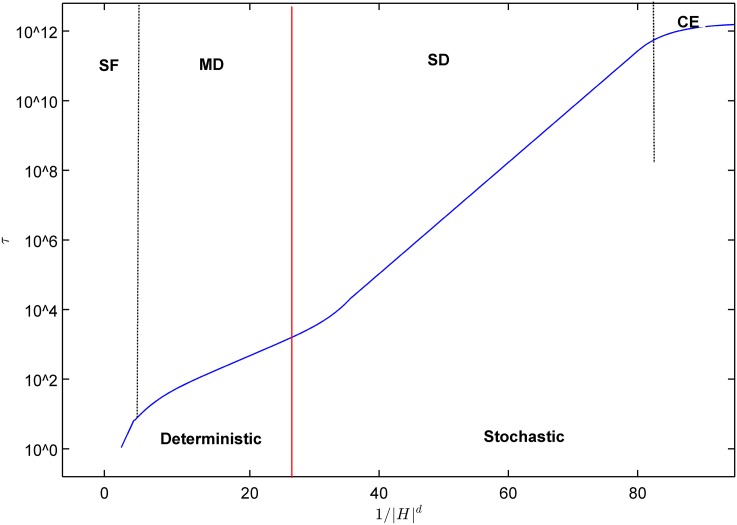
The schematic life time of metastable states as a response to the strength of the stimulating field from [14]. The weaker the stimulating field, the longer the life time. The first two regimes on the right are stochastic regimes in which the life time is not only long but also has serious variations. These two regimes are named as the coexistence regime and the single droplet regime. For stronger fields we have two regimes in which transition from metastable states happens in a deterministic way with a small variation. These two regimes are called multi droplet and strong field.

Generally, the size of the system, its temperature, and the intensity of the stimulating field can influence the lifetime of the metastable states. Most studies in cited papers take the temperature around 0.8*T*_*c*_. When we study the response of the system to the stimulating field we basically define four separate regimes. The lifetime of metastable states has been depicted schematically in Fig 3, see [14] for more details.

Let’s explain what Fig 3 represents. At the right hand side of the graph we show the responses of the system to very weak fields. When the stimulating field is too weak the lifetime of the metastable states can be dramatically high. For finite size systems in this regime, the model lives in a coexistence regime in which thermal fluctuations are even more important than the stimulating field. In this regime even if the system moves upward, although thermal fluctuation dipoles may move downward again, the regime is however a stochastic regime in which the variance of the lifetime is comparable with its mean value. Both the magnitude of the lifetime and its variance depend on the size of the system, which dramatically grows as the size grows. Such a regime is called a “Coexistence” regime.

The second regime is called the single droplet regime. In this regime the stimulating field is a little bit stronger, and the system would finally go to its global minimum. The lifetime is however long and the response of the system is stochastic. We should wait for a chance for the formation of a droplet with upward spins. This droplet takes a long time to grow and capture the whole system. Although in this regime we can find an average value for the lifetime, the variance is still comparable with the average value. So, the regime is still a stochastic regime. This regime is called the “Single Droplet” regime.

The third regime is where the stimulating field is strong enough to form lots of droplets and grow in a manner to escape from the metastable trap in a predictable period. The lifetime is not stochastic anymore and would be

$$\begin{array}{c} \tau (H)=c_{1}e^{\frac{c_{2}}{3|H|}}{\left|H\right|}^{-5/3}, \end{array}$$ (2)

in which *c*_1_ and *c*_2_ are constants. As we expect, the lifetime dramatically decreases as the stimulating field increases. This regime is called the “Multi Droplet” regime.

The last regime is called the “Strong Field” regime. It is a regime in which the stimulating field is strong enough to allow each spin to have a chance to flip. The rate is so high that we do not need to wait for some survivable droplets to form.

## Results

### Relation Between Macroeconomic Variables and the Ising Model

In the previous section we reviewed the responses and the lifetime of metastable states in the Ising model. We now need to interpret these responses in the language of economy. We need to report a minimum bound and money value of a fiscal stimulation. For the beginning let’s consider a cubic *D* dimensional lattice. In *D* dimensions each dipole has 2*D* neighbors. In a downward vacuum their force on a dipole is around −2*DJ*, see Fig 2. If they all turn from a downward direction to an upward direction, their force on our dipole is equal to 2*DJ*. Our stimulating field appears as *H*. In a downward vacuum each spin feels a downward force from neighbors almost equal to −2*DJ*, where if the stimulating field is about 4*DJ* each spin feels a net force equal to 2*DJ*. In this case, each spin can simply suppose that all of its neighbors are upward and there is no exogenous field.

On the economy side we supposed that the transfer of goods or money between firms forces them to chose a downward or upward direction. Suppose that in an expansion when all firms and corporations work with maximum capacity, the GDP is in its maximum level which we denote as *GDP*_+_. In a recession when all firms work with their minimum capacity, the GDP is in *GDP*_−_ level. Let’s denote the difference between this gap as Δ*GDP* = *GDP*_+_ − *GDP*_−_. In a recession, when all firms work with a minimum capacity, if the government has a purchase as big as Δ*GDP*, then it has compensated for the reduction of purchase by its neighbors. If we compare it with our Ising model, we find that a stimulation as big as Δ*GDP* resembles a stimulating field as big as 4*DJ*.

Stimulating bill may be designated to be spent for periods more than one year. In this case we need to write

$$\begin{array}{c} \frac{bill}{\Delta GDP}=\frac{\tau HN}{4DJN}=\frac{\tau H}{4DJ}\;\;\Rightarrow \;\;bill=\frac{\tau H}{4DJ}\Delta GDP, \end{array}$$ (3)

where *N* is the number of spins in the lattice or the number of nodes in a network, and *τ* is the time interval or Monte Carlo steps that we stimulate the Ising model.

A point that should be noted is that our transformation of parameters for the Ising model to the macroeconomics variable is true if we suppose that the firms decide for their production level on an annual base. Actually, lots of contracts with labors are on annual basis. This however by itself does not mean that firms change their strategies on an annual basis since contracts of different labors may be expired in different months. Strategies however will not change with a monthly basis. Firms can bear with fluctuations of a couple of months with their inventory investments. This discussion however is of importance in the interpretation of our findings. In the end of the paper we will recall this matter and discuss the robustness of our findings for this interpretation. For now let’s suppose that firms change their strategies on an annual basis.

In metastable studies the strength of the stimulating field has been discussed. In economy however we are mainly concerned with the budget. So, not only the strength of stimulation in each year, but also the period in which this stimulation should be imposed is important. As a result, the term that is important for us is *τH* rather than *τ* or *H*. If there is a minimum bound for the term *τH* to move an Ising model from one of its vacuums to the other, it means that for our network of firms there is a minimum bound for an effective stimulation. So, we seek to find the minimum of *τH* in the Ising model.

As discussed in the previous section, to classify the lifetime of the metastable states we have four different regimes. The first two regimes where the stimulating field is too weak are not of our interest. This is because the lifetime is stochastic. Neither politicians, nor policy makers are interested in a stimulation that has a stochastic lifetime. Politicians will loose their positions and policy maker will loose their reputation. The first goal of a stimulation is to trigger economy from recession to an expansion in a proper period of time. So, we leave the stochastic regimes in Fig 3 aside and focus on Multi-Droplet and Strong-Field regimes.

In the Multi-Droplet region the lifetime has been indicated in Eq (2). Our aim is to find the minimum of *τH* or $c_{1}e^{\frac{c_{2}}{3|H|}}{\left|H\right|}^{-2/3}$. This function is clearly a decreasing function of *H*. So, the bigger the magnitude of the magnetic field, the smaller the *τH*. So, to find a minimum for *τH* we are forced into the Strong-Field region.

Response of an Ising model to a strong magnetic field has been discussed in [18] and [19]. The lifetime has been of interest in the mentioned references. Since we need to find the minimum of the term *τH* rather than *τ* we need to run our own simulations.

**Fig 4:**
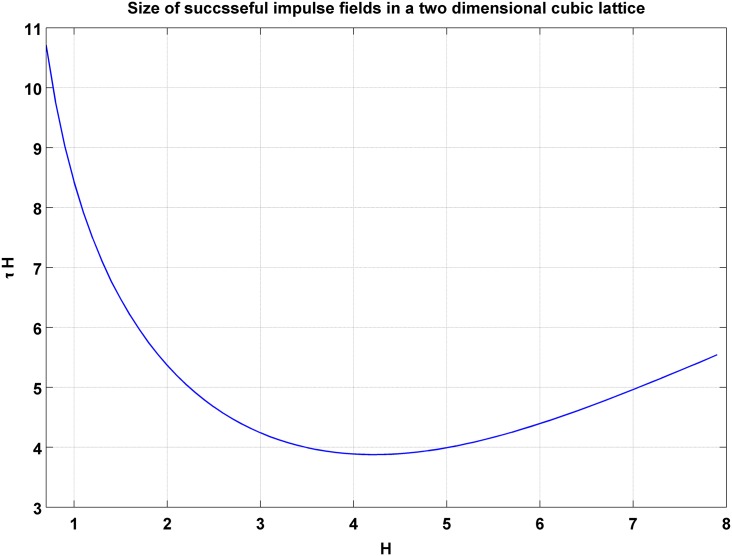
The result of our simulation for finding *τH* for metastable states has been graphed. H is in units of J (the coupling constant). The model is a two dimensional 1024*1024 lattice. The current figure is for one iteration. The minimum of *τH* is obtained (3.877 ± 0.004)J for 100 iterations.

We considered a two-dimensional 1024*1024 cubic lattice at *T* = 0.8*T*_*c*_ with a system living in its downward vacuum. We then imposed a magnetic field and updated each spin through the Monte Carlo method with the Glauber weight. We draw *τH* as a function of *H* in Fig 4. This figure deserves attention; first of all both strong and weak fields result in a higher value of *τH*. It is clear why weak fields result in higher rates of *τH*. This is because for weak fields the chance for every spin to flip is small. Strong fields as well result in higher values for *τH*; the reason for this is that to flip every spin we do not need a field stronger than the neighboring effects which is equal to 4*J*. The minimum of the curve however is obtained for *H* ≈ 2*DJ*. This means that for a minimum stimulation, the government needs to impose incentives for corporations with a rate that compensates for half of the reduction of orders in the recession. In the minimum, we have *τH* = (3.877 ± 0.004)*J*. Such a field suggests a minimum for fiscal stimulation equal to 0.48Δ*GDP*. For such stimulation, before we update each site once in an average, the magnetization tends to zero. In other words, the lifetime is below one Monte-Carlo step.

### Studying Small World Network

**Fig 5:**
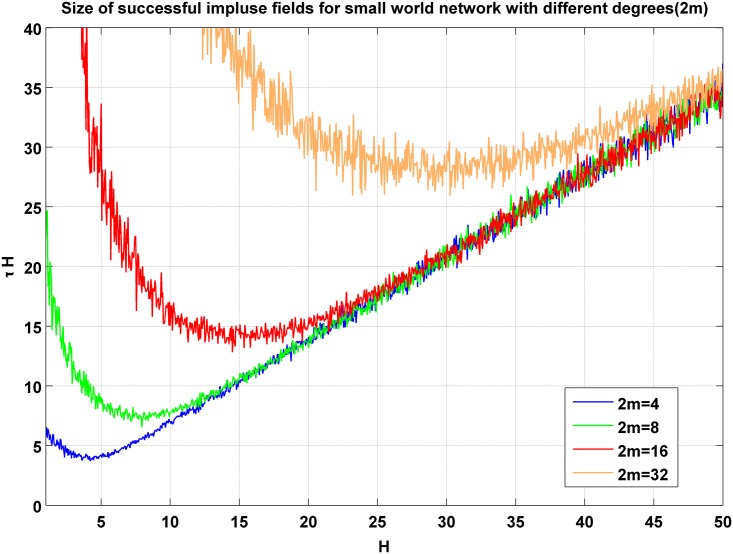
The result of simulation for Watts-Strogatz small world. As long as the degrees grows, the minimum for *τH* grows.

**Fig 6:**
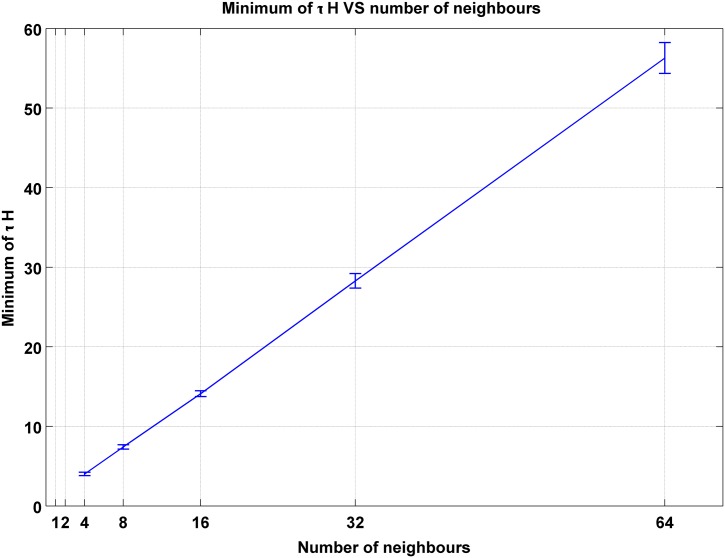
The minimum of *τH* grows linearly with the degree of nodes. So, the ratio of *τH*/2*mJ* tends to an almost constant rate.

A regular cubic lattice for the network of firms is applicable for a very simplified world. A better approximation can be reached via going to a more realistic network. In this line we focus on Watts-Strogatz network. We consider networks with 2*m* neighbors and let *m* vary from 2 to 32. The result of this simulation is depicted in Fig 5. As it can be seen in the figure, as the degree of nodes grow, the minimum grows accordingly. Minimums of *τH* as a function of *m* has been depicted in Fig 6. It can be seen from the figure that the minimum of *τH* grows linearly with *m*. This is a good news for us. If we look at Eq (9), we notice that a linear growth of the minimum of *τH* means a unique answer for a suggested bill. This means that to find a minimum for the stimulation policy we do not need to know the degree distribution of the network as long as it is a Watts-Strogatz one. Our suggestion for the minimum of the stimulating bill will be robust against the variation of the degree as long as the variation of degrees is not divergent. For large *m*, the minimum merges to 0.878 ± 0.027. So, the minimum bound for stimulation is *bill* = (0.439 ± 0.013)Δ*GDP*.

**Fig 7:**
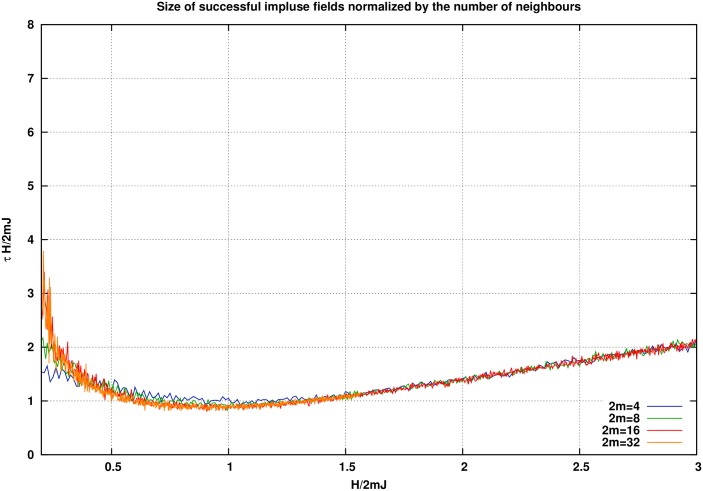
If in a small world we re-scale the curves with the degree of nodes, then all curves fit together. This means that all networks suggest the same minimum bound for the stimulating field.

Actually if in Fig 5 we re-scale each curve by the degree of the network or *m*, the results give the curves of Fig 7. As it can be seen, all curves are unified through this scaling. Let’s see how it happens. In the strong field regime where we impose an intense stimulating field, after only a few Monte Carlo steps the system moves from one of the metastable states to the other. This means that with such strong fields, each node does not have enough time to be influenced by the far nods. Only close neighbors are important. In this case the mean field approximation works properly.

Let’s suppose that in our network each node in average has 2*D* neighbors. For dynamics of magnetization we can write

$$\begin{array}{ccc} \frac{\partial m}{\partial t} & = & \frac{1}{2}[-m+\tanh\left[-\frac{1}{K_{B}T}\left(H-2mDJ\right)\right]\\ & = & \frac{1}{2}[-m+\tanh\left[-\frac{2DJ}{K_{B}T}\left(H/2DJ-m\right)\right] \end{array}$$ (4)

Since we have supposed that *T* = 0.8*T*_*c*_ where *T*_*c*_ is proportional to *D*, Eq (4) reduces to

$$\begin{array}{c} \frac{\partial m}{\partial t}=\frac{1}{2}\left[-m+\tanh\left[-C\left(H/2DJ-m\right)\right]\right], \end{array}$$ (5)

in which C is independent of the dimension or degree of nodes. Now, if we re-scale H as *h* = *H*/2*DJ*, the equation reads as

$$\begin{array}{c} \frac{\partial m}{\partial t}=\frac{1}{2}\left[-m+\tanh\left[-C\left(h-m\right)\right]\right]. \end{array}$$ (6)

As it can be seen after re-scaling *H* by a factor of 2*DJ*, we find a unique equation for its dynamics. This is why in Fig 7 after re-scaling the size of the stimulation by the degree of nodes we find a unique curve. So, the minimum of *τH* grows with the average of the degree of nodes.

Our observation implies that in Eq (9) the size of the minimum successful bill is universal. In other words it is independent of the number of neighbors or degree average of nodes. We however should be careful since in our mean field argument we supposed that we could work with the average of neighbors. Our proof holds true if the standard variation of the degree of nodes is small in respect to its average. If we are working with scale free networks then we need to be more careful. We have omitted such cases at the current level.

**Fig 8:**
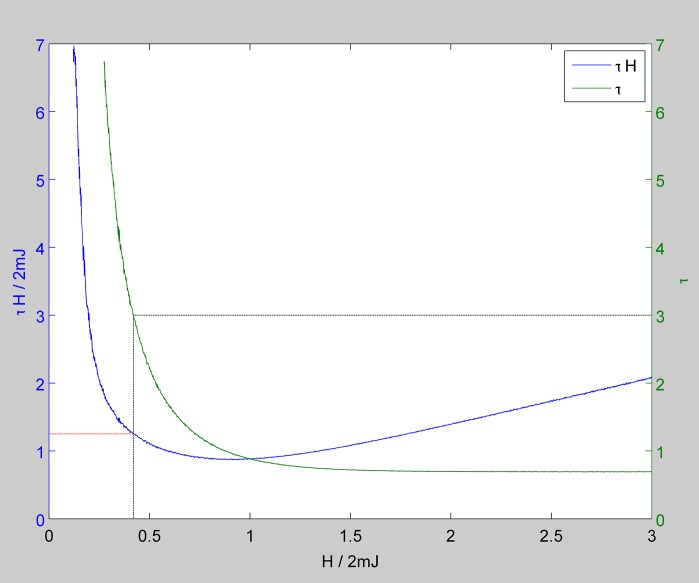
In this figure we have graphed both the average of *τH* and *τ* for fifty iterations for a Watts-Strogatz network with degree of 16. Such a graph comes handy for the case that we have limits for the budget in each year. If we aim to stimulate the market in periods longer than one step, then we can work with this figure in an Ising simulation of the network of firms. If for example instead of one Monte Carlo step we aim to stimulate the market in three steps, then it means that the size of *H* is about 0.42. This strength suggests the value of 1.25 for *τ* be equal to *H*. This means that in this case, the total bill is about 0.62Δ*GDP*. So for a three year stimulation, in each year we need a stimulation around 0.21Δ*GDP*. For a two-year spending stimulation we need about 0.53ΔGDP for the whole bill and 26%Δ*GDP* for each year.

**Budget Constraint:** If a government does not have enough budget to impose a big stimulation in one year, although the annual spending can be lower, the whole spending should be higher. In Fig 8 we see both *τH* and *τ* in terms of the strength of stimulation. The lowest budget can be effective when stimulation is imposed for only one period which is 0.44 of the GDP gap. If the government has a budget constraint and is willing to stimulate the economy for more than one period, then this figure comes handy. The green curve shows the period that a stimulation needs to be imposed and the red curve shows the value of the total stimulus bill. If for example the government aims to stimulate the economy in 3 years, then the value of stimulation would be about 0.64Δ*GDP*, and if we have a two year plan, the minimum bound would be around 0.54Δ*GDP*.

### Results for the Great Recession

In this section we aim to find what our model suggests for the minimum stimulation for the United States. However finding a minimum is a bit hard. This is because in the year 2009 when the GDP was declining, stimulation was imposed. So, in one hand we had an avalanches of defaults and on the other we had stimulation being imposed. As a result extrapolating an exact value for the real gap is not possible. We however try to do our best and have at least a rough guess for the minimum bound based on our analysis.

To extrapolate the gap of GDP for the United States we considered the time series of the real growth rate between the years 2000 and 2007. A linear extrapolation suggested a real growth around 2.5% for 2008 and 2009. In spite of this 5% growth, the economy of the US declined about −0.3 in 2008 and −2.8% in 2009. So, a rough guess for the gap is 8.1%. The major part of Obama’s stimulus package or *“The American Recovery and Reinvestment Act of 2009”* was spent in the years 2009 and 2010. The minimum size suggested by our model for a two year period according to Fig 8 is around 0.54Δ*GDP*. The GDP of the US in 2007 was around 14938 billions of 2009 US $. So, our model suggests a minimum bound for stimulation around *bill* = 0.54 ∗ 0.081 ∗ 14938 × 10^9^$ ≈ 650 billion $.

Obama’s stimulation policy bill *“The American Recovery and Reinvestment Act of 2009”* was firstly 787 billions. Later the bill was revised to 831 billions. The bill was to be spent between the years 2009 and 2019. The major part however was spent during 2009 and 2010. As of the second quarter of 2010, about 480 billions had been spent. As of the first quarter of 2011, around 670 billions had been spent [37]. So, the spent bill for the first two years would be a bit over our threshold.

If we repeat such analysis for the European Union we reach a minimum bound around 4.7% of GDP. This is while *“The European Economic Recovery Plan”* stimulus bill was around 1.5% of GDP.

In the US, in the first quarter of 2009 according to the OECD database, unemployment was about 8.27%. It reached its peak in the forth quarter of 2009 equal to 9.93%. Then it started to reduce where in the second quarter of 2011 reached 9.07%. So the bill was successful in reversing the growth trend of unemployment and reduce it from its peak by 1%. The major part of the bill was spent through 2009-2010. Part of the bill however was spent in the years after. Unemployment kept its decreasing trend where the US finally managed to overcome the recession. In the EU things went in different ways. In the first quarter of 2009 unemployment was 8.27% reaching its local peak by the first quarter of 2010 equal to 9.56%. The bill however was not able to reverse the growing trend substantially. It experienced its lowest rate in the first quarter of 2011 which was 9.39% which was only 0.3% below the peak. Unemployment then grew gradually and the EU could not overcome the recession.

For sure there has been a wide range of policies affecting economy. Extensive monetary policies held by the Central banks are of the most serious. To have a reliable conclusion one needs to consider a careful temporal pattern of spending in a number of countries. Still a conclusive conclusion can not be reached unless taking in to account effects such as monetary stimulation. So, we can not attribute the recovery of the US economy totally to the fiscal stimulation act. Nevertheless the stimulus act has been of great importance. Based on our simple model the US stimulus package was above the minimum barrier of the metastable feature of the network where the European one was far below it. So, at least at the first level of approximation, our model provides a successful prediction for the two major economies of the world.

## Discussion

Considering an Ising model for the network of firms is simplifying the real world. It however sheds light on our problem and exposes existence of a minimum bound for stimulation. If the network of firms is a Watts-Strogatz network then our result is independent of the degree of nodes.

**Short range or long range interaction?**

One point of strength of our method is that it lives in a regime described by the mean field. If we look at Eq (5) we notice that it has deep consequences. Keynesian economics believes in animal spirit. When economy falls into a deep recession, people themselves cut their consumption and hesitate risky movements. Such effect makes the crisis more disastrous. Getting out of recession will be even harder. If a stimulus bill triggers aggregate demand, as long as unemployment decreases, more and more people start to expand their consumption. At first it seems that since in an Ising model we have considered only the effect of neighbors, then the global situation has been neglected. To state clearer, one may think that since in a Keynesian economics, global scheme of economics affects decisions of each individual, the long range interaction of forces must be considered. The point is that in a mean field regime, as it can be deduced from Eq (5), when *m* tends to −1, the growth rate as a response to the stimulating field decreases substantially. This means that despite our approximation of the short range interaction, the response to stimulation captures global scheme.

**Variation over temperature:**

The role of temperature deserves more attention. There is no clear understanding about temperature in a many body system such as economy where there might be different intuitions. Yakovenko and Rosser for example have suggested the average of GDP per capita as temperature [38]. Any claim about a general definition of temperature in economy may raise some debates. In simple models however we can discuss the role of temperature. In our work we have tried to model network of firms by an Ising model. In an Ising model temperature states the level of correlation between neighboring spins. The chance for two spins to have a different direction is given by

$$\begin{array}{c} p\propto e^{-\frac{2J}{KT}}. \end{array}$$ (7)

If you have *m* neighbors all choosing to decrease their production; the chance that one keeps a high level of production is

$$\begin{array}{c} p\propto e^{-\frac{2mJ}{KT}}. \end{array}$$ (8)

If we suppose the temperature to be very small, it means that if neighbors of a firm choose to cut their production, the firm itself would definitely cut its production. It is not however the case in the real world. Such assumptions ruin the stochastic structure of decisions made by a manager as well as local fluctuations of the market. So for sure, to consider stochastic features of the market we can not set the temperature close to zero.

Now, the question is weather if we can choose a relatively high temperature to model network of firms. Actually as we have stated in the introduction, if you reason within a Keynesian framework, the temperature should be below the critical point. In this framework, it is believed that in a recession, without government intervention the life of a metastable state might be very long, and the market may live in a long recession. So, if we aim to model an economic network with an Ising model in a Keynesian framework, to have metastable features, the temperature should be below the critical point. Besides, if we look at the unemployment time series of the US, we observe that economy is living in periods of high and low unemployment rates. In a work presented by Safdari et al. [39] it has been shown that fractal dimension of unemployment time series is 0.66. So, it means that unemployment possesses memory and actually has a positive correlation with its past. In hot temperatures in the Ising model, magnetization has no memory and fluctuates around zero. As a result, in an Ising model of economy temperatures can not be very high. So, a moderate temperature below the critical point is reasonable.

**Fig 9:**
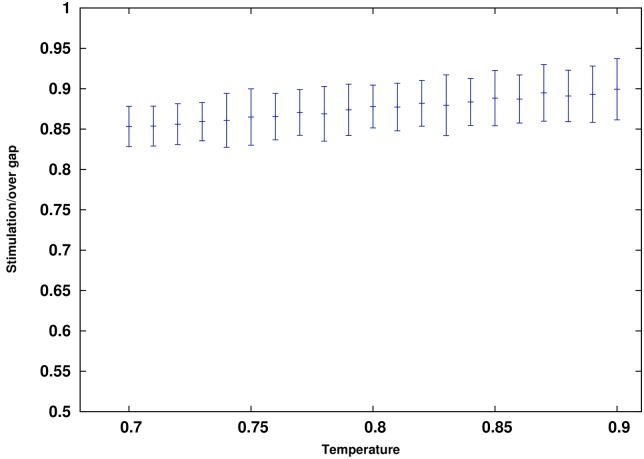
The minimum of *τH* over different temperatures range from 0.7*T*_*c*_ to 0.9*T*_*c*_. Network is a Watts-Strogatz with degree of 16 and *P* = 0.01. As it can bee seen within a reasonable window of temperature, our result does not vary considerably.

Now, still below the critical point, one might be concerned about the vulnerability of our results on the temperature. We ran our simulations in *T* = 0.8*T*_*c*_. In the introduction we have justified that if we aim to model the economy within the framework of the Keynesian economics we need to suppose that temperature is below the critical point. As far as we are concerned with qualitative discussion and metastability features, the exact temperature does not matter. In our discussion however we have translated our results into quantitative measures in economics and thereby need to check the vulnerability of our results on the temperature. In this regard we reconsidered our small world model with *K* = 16 and repeated our simulation for a range of different temperatures. Results based on an ensemble of 50 iterations has been depicted in Fig 9. As it can be seen in a reasonable regime of temperature, our result is robust and does not vary considerably.

### The Golden Time Passage

Our study notified the metastable feature of the market. It is a prisoner dilemma. In recession, if a major portion of employers hire more labor and increase their production, the hired labors will buy more and everybody including managers are better off. If however only a small portion of firms do this, they will be losers. So, in spite of the global benefits, local interests prevent the market from reaching its best situation. However as time goes by, a shock such as a technological shock or demand shock may trigger the market to a global extremum, but it would be time consuming. For the Great Depression, the World World War II rose such a demand. A fiscal expansionary policy aims to stimulate a major portion of firms to rise their production and recover the economy in a reasonable period. We found that there was a minimum bound for a successful stimulation. Our results are however vulnerable to the interpretation of the Monte Carlo steps to the real time. To reach Eq (9) we considered a Monte Carlo step as of one year.

Actually if policies of managers are changed within a period of *T*, we need to modify Eq (9) to

$$\begin{array}{c} bill=\frac{\tau H}{4DJ}T\Delta GDP, \end{array}$$ (9)

in which *T* is expressed by the unit of year. If managers decide to cut or rise their production quarterly, the minimum bound would be one fourth of our previous suggestion. The point is that in a deep recession, if an employer has fired part of her employees then it would be unlikely that she changes her mind easily. Even if for a few months the orders grow, a manager may hesitate to employ new labors. If decline in production is due to decrease of working hours, however, then it will be much easier for managers to rise their production. So, when we want to translate the result of our simulation to the language of economy, we need to be clear about the situation. In the case that only working hours have been decreased, the minimum bound for a successful stimulation can be much lower as long as the stimulation is shovel ready. In that case, managers can easily change their mind and rise working hours. So, an update in a Monte Carlo step, or actually an update for a new level of production for each agent can happen in a much shorter time. As a result, the minimum bound would be much lower.

**Fig 10:**
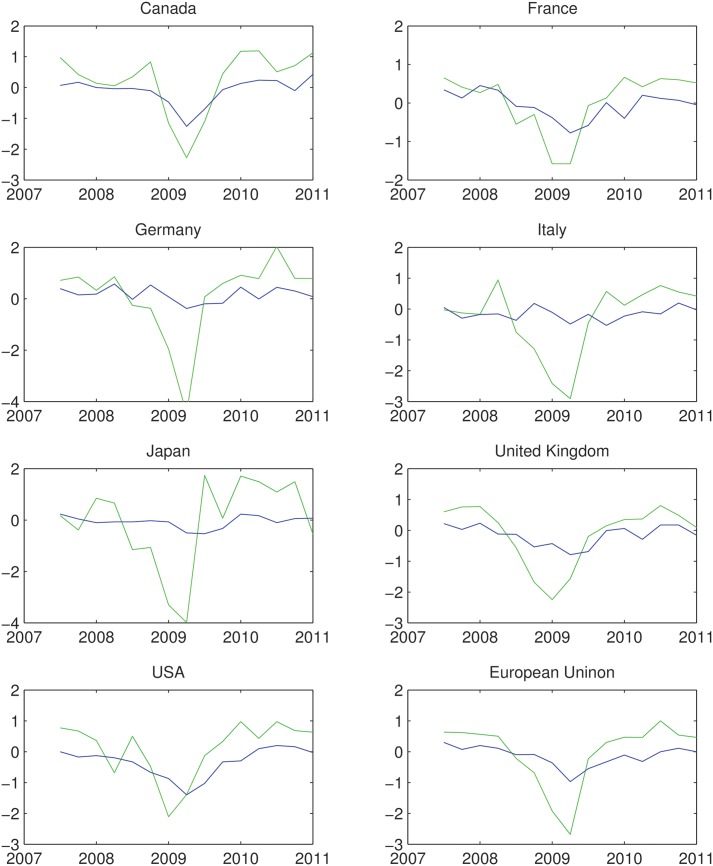
In this figure we have depicted both the changes in GDP and unemployment. The green curve shows quarterly growth rates of GDP. The blue curve shows the changes of unemployment in sequential quarters times −1. Unemployment and the GDP growth rate have negative correlation. Since we aim to show the lag between these two parameters at the time of crisis we multiplied the changes in unemployment by −1. As it can be seen at the time of crisis, the GDP declines before unemployment rises. The lag between the growth rate and unemployment is the golden time passage for a stimulation. Source: OECD.

If we look over the big economies through the years 2008 to 2009 we observe that the decline in the GDP growth rate starts a couple of months before the rise in unemployment, see Fig 10. In this figure we have depicted two time series. One of the time series is the GDP growth rate, and the other is the changes in unemployment times minus one. Changes in unemployment has negative correlation with the GDP growth rate under the Okun’s law. In this figure we however show that in the time of crisis this correlation has a lag. Since graphically it was easier to show the lag of our correlated parameters, we multiplied the variation of unemployment by minus one.

As it can be seen in the Fig 10, in almost all countries, GDP starts to decline a few months before unemployment starts to rise (or its negative form declines). The data from OECD has been rewritten in Table 1. In Germany for example during the forth quarter of the year 2008 and the first quarter of 2009, the GDP declined 6.4%. In the same period, unemployment raised less than half a percent. During the same period while the GDP had declined more than 3% in France, unemployment had grown less than 1.2%. In Japan as well, despite a decline around 6.7%, unemployment only grew around 0.5%. This observation is not surprising. This is due to the fact that on one hand lots of annual based contracts had already been signed, while on the other the everlasting attitude of managers in decreasing working hours before making decision and firing part of the employees existed.

**Table 1:** GDP growth rate and unemployment rate of G7 and the E.U. Source: OECD.

		2008I	2008II	2008III	2008IV	2009I	2009II	2009III	2009IV
Canada	Growth Rate	0.06	0.35	0.83	-1.16	-2.28	-1.10	0.45	1.18
	Unemployment	5.97	6.00	6.10	6.57	7.83	8.53	8.60	8.47
France	Growth Rate	0.48	-0.55	-0.30	-1.58	-1.58	-0.07	0.13	0.67
	Unemployment	6.84	6.93	7.05	7.43	8.21	8.79	8.78	9.18
Germany	Growth Rate	0.85	-0.26	-0.37	-1.96	-4.45	0.07	0.59	0.91
	Unemployment	7.92	7.95	7.42	7.35	7.73	7.93	8.11	7.66
Italy	Growth Rate	0.94	-0.75	-1.29	-2.42	-2.91	-0.45	0.57	0.12
	Unemployment	6.51	6.88	6.70	6.81	7.30	7.47	8.00	8.23
Japan	Growth Rate	0.66	-1.15	-1.07	-3.30	-3.39	1.74	0.07	1.71
	Unemployment	3.90	3.97	4.00	4.07	4.57	5.10	5.43	5.20
UK	Growth Rate	0.06	0.35	0.83	-1.16	-2.28	-1.10	0.45	1.18
	Unemployment	5.14	5.27	5.81	6.24	7.03	7.72	7.73	7.67
US	Growth Rate	0.25	-0.56	-1.68	-2.25	-1.57	-.20	0.15	0.35
	Unemployment	5.00	5.33	6.00	6.87	8.27	9.30	9.63	9.93
EU	Growth Rate	0.50	-0.22	-0.68	-1.92	-2.68	-0.23	0.30	0.47
	Unemployment	6.75	6.85	6.94	7.30	8.27	8.82	9.15	9.26

Now, we notify that our observations on metastable features suggests a golden time passage for a fiscal stimulation. This time passage is the period that after a shock such as the bubble burst of 2008 firms abruptly reduce their production as a precautionary act for decline in orders. Besides, people as a precautionary act reduce their consumption. This is while firms still have not fired their employees. Since during this time passage re-rising production for managers is much easier, and a Monte Carlo step means a shorter time in the real world, a minimum bound for a successful stimulation is much lower. A rough estimation from the time series in Fig 10 suggests that this time passage could be around two quarters. So, a serious lesson from studying the metastable feature is that when a shock such as the 2008 crash irrupts making us to expect a recession, we better hurry to impose stimulation instead of sitting for negotiations in congress and offices. In that case a much smaller stimulation may prevent economy from falling into a deep recession. In this time passage every single day counts. As time goes by the minimum bound for a successful stimulation rises. We should notice that at the time of crisis we have a percolation of the crisis between different sectors of the market. Such percolation itself emphasizes a fast response. Our observation however suggests that if the imposed simulation takes place fast, even a small fiscal stimulation might prevent the recession to percolate. The efficiency of the stimulation is highest in the period between two acts; the cut down in working hours and the resulting labour firing. A relatively small but very fast stimulation before the second act is more effective than a pretty large stimulation after the firing process.

## Conclusions

Keynes has claimed that in the time of crisis, the animal spirit forces people to cut consumption. The consumption behavior is a key debate between the two mainstream schools of economics. Our work however emphasizes a totally different aspect of economy. We suggest the intervention of governments due to the problems other than the consumption behaviour and animal spirit. We mean that whatever the end of the chain consumers do; cut or uncut their consumption, in the intermediate level, network of firms and corporations have their own problems. They can not look at aggregate indexes before cutting or increasing their production. If unemployment decreases in Florida and some other east states, service providers can not hire new labors in e.g. California. Each firm or corporation should look at its neighbors in the network and decide whether to hire new labors or not. This results in the occurrence of a metastable behavior in the network of production. Nonetheless, there exists friction for finding new connections. To state clearer, when you just need to play with local connections in your network, metastable features emerge as an inevitable result.

Keynesian economists favor fiscal and monetary stimulation for recovery. Our analysis of metastable features of the network, however suggests that if policy makers decide to impose a demand shock, then there is a minimum bound for such a stimulation to be successful. However by working with a simple model, our results furnish a sense of reality though rough at the current stage. We provided an early guess for this minimum bound based on the depth of the crisis and the production gap. Our suggestion is valid as long as the network of firms is Watts-Strogatz. Since the network of firms shows scaling degree distributions [40], a future work on a more realistic network would provide a better understanding regarding the response of the network of firms and corporations.

In the United States, the stimulus package was around our minimum bound where the US managed to overcome the recession. In the European Union, the stimulus package was far below our minimum bound where the European Union was unable to fully overcome the crisis. Although having two successful predictions is not a noticeable confirmation of an analysis, it however is satisfying at the first level of examination. A thorough examination needs a careful analysis of the temporal pattern of spending in different countries which can provide motivation for a further study.

In this work we implement a simple model. Nonetheless, in the future, we should develop the model to provide a more accurate resolution on the matter. Currently evaluating the response of the market to stimulation is being studied from different point of views. In an interesting work for example, Gallegati, Landini, and Stiglitz have shown that inequality has an adverse effect on the multiplying factor of the market [41]. So, it is expected that in the future, through studies of the collective behavior from different point of views, we obtain better resolution on the response of the network of agents regarding the government role.

In this work, for simplicity we have supposed that nearly all firms work with maximum capacity in times of expansion, and a minimum capacity in times of recession. This is not the case in the real world. In a recession, still a portion of firms work with maximum capacity. Besides, firms are not bound to have a binary choice. They can decrease their production in different levels. So, a Potts or XY model may describe the case more realistic, which can be of a future work. In addition a spin glass explanation can let different vacuums for the model. All of these extensions however bring more complications to the model as they improve the results heterogeneity.

One of the most important findings of our analysis is the suggestion of the golden time passage. Although everybody has an intuition that at the time of crisis an early response by the government helps preventing the propagation of crisis, our analysis reveals another important point. Our analysis reveals that the pattern of making decision by individuals has a serious impact on the minimum bound of the successful stimulation. In the network language, the response is normalized by Monte-Carlo time intervals. As a result, if we are sure that the cascade is not going to spread any more, still there would be a serious difference between a situation where only working hours have been declined in the firms and a situation where labors have been fired. In the former situation a minimum bound for a successful stimulation is much lower. It in some senses resembles hysteresis. Before the system relaxes to the new level of supply and demand thresholds, reversing the effect would cost much less.
